# Picolinic Acid-Mediated Catalysis of Mn(II) for Peracetic Acid Oxidation Processes: Formation of High-Valent Mn Species

**DOI:** 10.1021/acs.est.3c00765

**Published:** 2023-05-24

**Authors:** Juhee Kim, Junyue Wang, Daniel C. Ashley, Virender K. Sharma, Ching-Hua Huang

**Affiliations:** † School of Civil and Environmental Engineering, Georgia Institute of Technology, Atlanta, Georgia 30332, United States; ‡ Department of Chemistry and Biochemistry, Spelman College, Atlanta, Georgia 30314, United States; § Department of Environmental and Occupational Health, School of Public Health, Texas A&M University, College Station, Texas 77843, United States

**Keywords:** peracetic acid, picolinic acid, high-valent Mn species, micropollutants, wastewater treatment

## Abstract

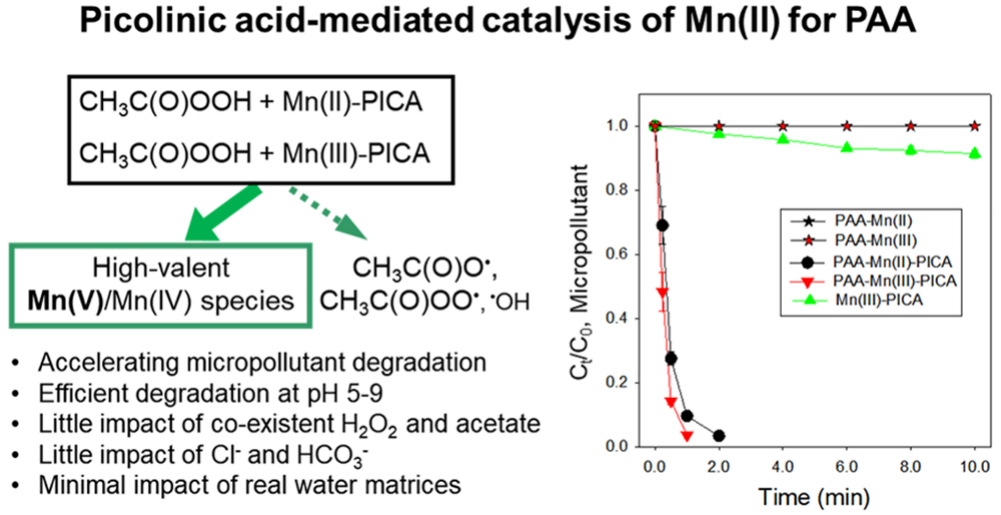

Metal-based advanced oxidation processes (AOPs) with peracetic acid (PAA) have been extensively studied to degrade micropollutants (MPs) in wastewater. Mn(II) is a commonly used homogeneous metal catalyst for oxidant activation, but it performs poorly with PAA. This study identifies that the biodegradable chelating ligand picolinic acid (PICA) can significantly mediate Mn(II) activation of PAA for accelerated MP degradation. Results show that, while Mn(II) alone has minimal reactivity toward PAA, the presence of PICA accelerates PAA loss by Mn(II). The PAA-Mn(II)-PICA system removes various MPs (methylene blue, bisphenol A, naproxen, sulfamethoxazole, carbamazepine, and trimethoprim) rapidly at neutral pH, achieving >60% removal within 10 min in clean and wastewater matrices. Coexistent H_2_O_2_ and acetic acid in PAA play a negligible role in rapid MP degradation. In-depth evaluation with scavengers and probe compounds (*tert*-butyl alcohol, methanol, methyl phenyl sulfoxide, and methyl phenyl sulfone) suggested that high-valent Mn species (Mn(V)) is a likely main reactive species leading to rapid MP degradation, whereas soluble Mn(III)-PICA and radicals (CH_3_C(O)O^•^ and CH_3_C(O)OO^•^) are minor reactive species. This study broadens the mechanistic understanding of metal-based AOPs using PAA in combination with chelating agents and indicates the PAA-Mn(II)-PICA system as a novel AOP for wastewater treatment.

## Abstract 2

Peracetic acid-Mn(II) in combination with picolinic acid generates high-valent Mn species (Mn(V)) that contributes to rapid degradation of micropollutants with minimal influence from water matrices.

## Introduction

Peroxyacid peracetic acid (PAA, CH_3_C(O)OOH) is an emerging oxidant/disinfectant that has been applied in various industries, including municipal wastewater treatment, food processing, pulp and paper, and medicine.^1−7^ With several advantages over the conventional chlorine oxidants, including high disinfection efficacy, low toxicity to mammals, and much less formation of toxic byproducts,^8,9^ the application of PAA in wastewater and other industrial disinfection processes has been growing steadily.^10−16^ Recent reviews have reported that PAA-treated wastewater effluents have low ecotoxicological impacts, and PAA-based pulp bleaching generates little persistent toxic or mutagenic residuals or byproducts.^7,17^ Recently, PAA has received growing research attention as a promising oxidant in advanced oxidation processes (AOPs) for abatement of micropollutants (MPs) in wastewater.^18−29^ While PAA has relatively low reactivity toward MPs with the exception of sulfur moieties,^30^ PAA can be activated to produce highly reactive species that can more efficiently degrade MPs. Research has employed various activation approaches, including energy and catalysts, to produce highly reactive species (such as radicals of ^•^OH, CH_3_C(O)O^•^, and CH_3_C(O)OO^•^ as well as high-valent metal species when metal catalysts are employed).^21,22,27,28^ The ^•^OH is a well-known strong oxidant to degrade a wide range of MPs. CH_3_C(O)OO^•^ is also a powerful oxidant that rapidly reacts with MPs via electron transfer.^23,31^ CH_3_C(O)O^•^ has high oxidation power but is subject to rapid self-dissociation to less reactive ^•^CH_3_ and CO_2_ (*k* = 2.3 × 10^5^ s^–1^).^32^

Several metals, including Fe(II/III/VI), Co(II/III), and Ru(III), have been shown to be good catalysts for PAA activation.^20,24−26,33^ Only small amounts of those metals (10–200 μM) can efficiently activate PAA (100–200 μM) and degrade a wide range of MPs, including pharmaceuticals, antibiotics, and dyes. It is postulated that high-valent metal species are generated when metal catalysts are applied to activate PAA. High-valent metal species are known to be strong oxidants but with greater selectivity in reactivity than free radicals;^34−36^ thus, they may persist longer in environmental water matrices that contain various reactive species-scavenging constituents.

Manganese is a common transition metal found in natural environments and has a wide range of oxidation states from +2 to +7 in aqueous solutions. Water-soluble Mn(II) has been extensively studied as a homogeneous activator for many oxidants,^37−40^ but there is little information on PAA activation by Mn(II). To the best of our knowledge, only one study^41^ evaluated the abatement of MP by Mn(II)-PAA in aqueous samples. They reported that Orange II was degraded by Mn(II)-PAA at pH 9.4; however, a high dosage of PAA ([PAA] = 5–20 mM, [Mn(II)] = 100 μM) was required. Moreover, at acidic to neutral pHs (pH 3–7), the degradation of Orange II was limited in similar conditions. They proposed the formation of Mn(III) and Mn(IV), which was supported by UV/visible spectral changes and electron paramagnetic resonance (EPR) spectroscopy. It was also postulated that Mn(II) complexes with PAA to form the peroxo complex, which undergoes heterolytic cleavage to form Mn(IV)-oxo species and/or homolytic bond cleavage to yield Mn(III) and organic radical.

Catalytic activity of metals can be improved by in situ addition of some chelating agents. In our recent study, we found that picolinic acid (2-pyridinecarboxylic acid; PICA) is a highly efficient chelating agent that can accelerate the reaction of PAA with Fe(III), thereby facilitating the oxidation of MPs by the Fe(III)-PAA process at neutral pH conditions.^42^ PICA has been widely used as a chelating agent in chemical and pharmaceutical applications.^43,44^ Recently, it has been applied to water treatment, owing to its biodegradability and lower toxicity compared to other types of chelating agents.^42,45^ PICA, with an aromatic nitrogen atom and a carboxylate donor group, can form a five-membered chelate ring with a central metal ion.^46−48^ The σ-donor (and weak π-acceptor) features of the aromatic nitrogen of PICA boost the nucleophilicity of the metal center and the catalytic activity of the metal complex.^47^ Building upon the previous work, this study investigated whether PICA could improve the catalytic efficiency of Mn(II) for PAA oxidation processes in a wide pH range (pH 3.0–9.0). The study objectives included: (i) demonstrating the capability of the PAA-Mn(II)-PICA system to degrade a model MP, methylene blue (MB), under a wide range of reaction conditions (i.e., molar ratios of metal to ligand (Mn(II) to PICA), molar ratios of metal to oxidant (Mn(II) to PAA), solution pHs, and presence of other anions), (ii) identifying the generation of major reactive species and their contributions to MP degradation in the PAA-Mn(II)-PICA system by using scavengers and probe compounds, and (iii) investigating the abatement of various MPs (bisphenol A (BPA), naproxen (NPX), sulfamethoxazole (SMX), trimethoprim (TMP), and carbamazepine (CBZ)) by the PAA-Mn(II)-PICA system in clean and real water matrices (wastewater effluent) for the feasibility of this AOP.

## Experimental Section

### Chemicals

Sources of chemicals and reagents are provided in Text S1.

### Experimental Procedures

The Mn(II)-PICA solutions in various molar ratios ([Mn(II)]:[PICA] = 1:1–1:10) were prepared by adding 100 mM manganese(II) sulfate into the required quantities of PICA solution. The Mn(II)-PICA solutions were agitated in a rotary shaker for 60 min.

Experiments for oxidation of MPs by the PAA-Mn(II)-PICA system were conducted in 50 mL amber borosilicate reactors with continuous magnetic stirring ([PAA]_0_ = 100–500 μM, [Mn(II)]_0_ = 4.0–100 μM, [PICA]_0_ = 4.0–200 μM, [MP]_0_ = 15 μM). First, the reaction solution containing MP and oxidant (PAA or H_2_O_2_) was prepared in the amber borosilicate reactor, and the desired initial pH was adjusted by adding a few μL of NaOH (1.0 M) and/or H_2_SO_4_ (1.0 M) into the solution. The degradation of MPs by PAA alone or H_2_O_2_ alone (without Mn(II)) was negligible. Then, the reaction was started by adding a desired amount of Mn(II)-PICA solution, and sample aliquots were taken at regular intervals up to 10 min. The MB concentration was immediately determined spectrophotometrically at 665 nm (Beckman DU 520 UV–visible spectrophotometer, Beckman Coulter, Inc., Fullerton, CA, USA). For other MPs, the oxidant was quenched by adding 1.0 mL of sample aliquots into vials containing excess Na_2_S_2_O_3_ ([Na_2_S_2_O_3_]/[PAA]_0_ > 40). Samples were stored at 5 °C prior to analysis. The solution pH was measured again after the reaction (10 min), and pH decreased approximately 0.0–2.6 pH units from the initial pH of 3.1–9.0, respectively. The degradation of MPs by PAA-Mn(III)-PICA was evaluated with the same procedures ([PAA]_0_ = 500 μM, [Mn(III)]_0_ = 20 μM, [PICA]_0_ = 100 μM, [MP]_0_ = 15 μM) except that manganese(III) acetate dihydrate was used.

In the scavenging experiments to evaluate the influence of ^•^OH or high-valent Mn species, 50 mM *tert*-butyl alcohol (TBA), 50 mM methanol (MeOH), or 5.0 mM methyl phenyl sulfoxide (PMSO) was used. A reaction solution containing MP, PAA, and scavenger was prepared, and the reaction was initiated by adding a desired amount of Mn(II)-PICA solution. The conversion of PMSO to PMSO_2_ (methyl phenyl sulfone) was monitored in both PAA-Mn(II)-PICA and PAA-Mn(III)-PICA systems. Control experiments without PAA and/or PICA were conducted to evaluate the oxidation of MPs by Mn(II), Mn(III), Mn(II)-PICA, or Mn(III)-PICA. The effects of anions (chloride, bicarbonate, and phosphate) on the degradation of MPs by PAA-Mn(II)-PICA were also evaluated ([PAA]_0_ = 200 μM, [Mn (III)]_0_ = 20 μM, [PICA]_0_ = 100 μM, [MP]_0_ = 15 μM, [anion]_0_ = 10 mM, initial pH = 7.1). The initial pH was adjusted after adding the studied anions. The degradation of four MPs (MB, BPA, SMX, and NPX) was assessed in the tertiary effluent from a municipal wastewater treatment plant. The wastewater effluent contained about 0.5 mg/L NH_3_–N, 0.03 mg/L total-P, and 6 mg/L TOC, and pH was 6.5. Chemical properties of MPs and a probe compound are provided in Table S1. All experiments were conducted in duplicate, and average values were reported.

### Analytical Methods

The DPD method was used to determine the PAA concentration.^19,49^ The additional information on other analytical methods is available in Text S1.

## Results and Discussion

### Reaction of PAA with Mn(II)-PICA

**Figure 1:**
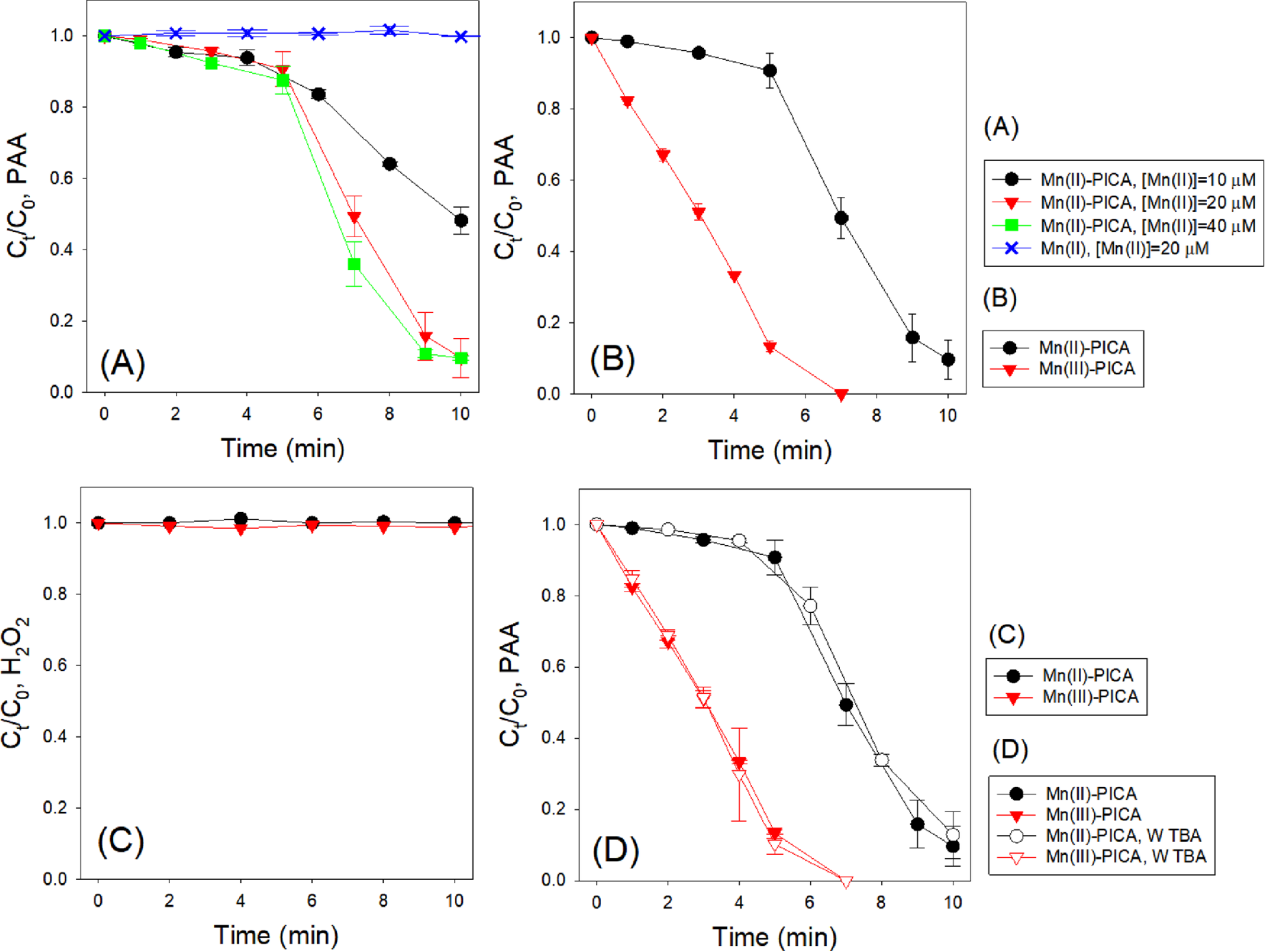
(A) PAA decreases by Mn(II) alone or by Mn(II)-PICA at different Mn(II) concentrations. (B) PAA decreases by Mn(II)-PICA or Mn(III)-PICA. (C) H_2_O_2_ decreases by Mn(II)-PICA or Mn(III)-PICA. (D) PAA decreases by Mn(II)-PICA or Mn(III)-PICA with and without TBA (conditions: initial pH = 5.0, (A) [PAA]_0_ = 200 μM, [Mn(II)]_0_ = 10–40 μM, [PICA]_0_ = 50–200 μM, [Mn(II)]:[PICA] ratio = 1:5; (B) [PAA]_0_ = 200 μM, [Mn(II) or Mn(III)]_0_ = 20 μM, [PICA]_0_ = 100 μM; (C) [H_2_O_2_]_0_ = 84 μM, [Mn(II)]_0_ = 20 μM, [PICA]_0_ = 100 μM; (D) [PAA]_0_ = 200 μM, [Mn(II) or Mn(III)]_0_ = 20 μM, [PICA]_0_ = 100 μM, [TBA]_0_ = 0 or 10 mM).

First, the decay of PAA by Mn(II) in the absence and presence of PICA was monitored (conditions: [PAA]_0_ = 200 μM, [Mn(II)]_0_ = 10–40 μM, [PICA]_0_ = 0 or 50–200 μM where [Mn(II)]:[PICA] ratio = 1:5, initial pH = 5.0). As shown in Figure 1A, the loss of PAA by Mn(II) without PICA was negligible. In contrast, the addition of PICA noticeably increased the loss of PAA, resulting in approximately 51.8–90.4% of PAA consumption within 10 min at increased [Mn(II)]/[PICA] concentrations, strongly suggesting that PICA enhances the reactivity of Mn(II) toward PAA. Importantly, the loss of PAA continued without adding additional Mn(II)-PICA (Figure S1), suggesting that reactive species (possibly intermediate Mn species) formed through the reaction of PAA with Mn(II)-PICA could further consume PAA rapidly.

Based on previous research on the reaction of PAA with metals such as Fe(II),^20^ Co(II),^24,29^ Ru(III),^26^ and Fe(III)-PICA,^42^ we proposed the following reactions in the PAA-Mn(II)-PICA system. In the presence of Mn(II) and PICA, the reaction of PAA with Mn(II)-PICA could form reactive species, such as Mn(III), Mn(IV/V), and/or CH_3_C(O)O^•^, according to eqs 1–4:
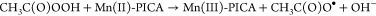
1
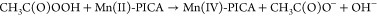
2
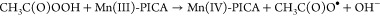
3
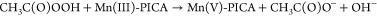
4Other possible reactions, generating ^•^OH and CH_3_C(O)OO^•^, may also be considered (eqs 5–8):
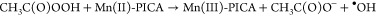
5
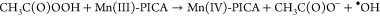
6
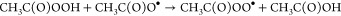
7
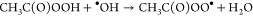
8

The possible decay of PAA by Mn(III)-PICA was then investigated separately (conditions: [PAA]_0_ = 200 μM, [Mn(III)]_0_ = 20 μM, [PICA]_0_ = 100 μM, initial pH = 5.0). As shown in Figure 1B, the rate of PAA loss was much faster when the reaction was initiated by Mn(III)-PICA. This result supports the above hypothesis that Mn(III)-PICA is likely an intermediate which has greater reactivity toward PAA. The fast reaction of Mn(III)-PICA with PAA could lead to the formation of higher valent Mn species, such as Mn(IV/V).

Since the PAA solution contained about 32% PAA, 6% H_2_O_2_, and 40% acetic acid, 83.8 μM H_2_O_2_ and 316.7 μM acetic acid were present in the 200 μM PAA solution. Reactions related to coexistent H_2_O_2_ might form reactive species (eqs 9 and 10):
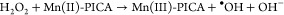
9
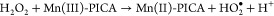
10However, we found that H_2_O_2_ did not decay by either Mn(II)-PICA or Mn(III)-PICA, whether or not acetic acid was present (conditions: [H_2_O_2_]_0_ = 84 μM, [Mn(II)]_0_ = 20 μM, [PICA]_0_ = 100 μM, initial pH = 5.0; Figure 1C). Additionally, we observed that the presence of additional H_2_O_2_ did not influence PAA decay by Mn(II)-PICA (conditions: [PAA]_0_ = 200 μM, [H_2_O_2_]_0_ = 184 μM (84 μM in PAA solution and additional input of 100 μM), [Mn(II)]_0_ = 20 μM, [PICA]_0_ = 100 μM, initial pH = 5.0; Figure S2). These results confirmed that coexistent H_2_O_2_ and acetic acid in the PAA solution played a negligible role in the overall reaction. Furthermore, the addition of TBA, a highly reactive quencher of ^•^OH (*k*_•OH/TBA_ = 3.8–7.6 × 10^8^ M^–1^·s^–1^),^50^ had a negligible effect on PAA decomposition by Mn(II)-PICA or Mn(III)-PICA (Figure 1D). As PAA is known to have high reactivity to ^•^OH,^19,51^ the little impact of TBA indicated that the generation of ^•^OH was likely negligible in the reaction of PAA with Mn(II)-PICA and Mn(III)-PICA.

### Degradation of MPs by the PAA-Mn(II)-PICA System

**Figure 2:**
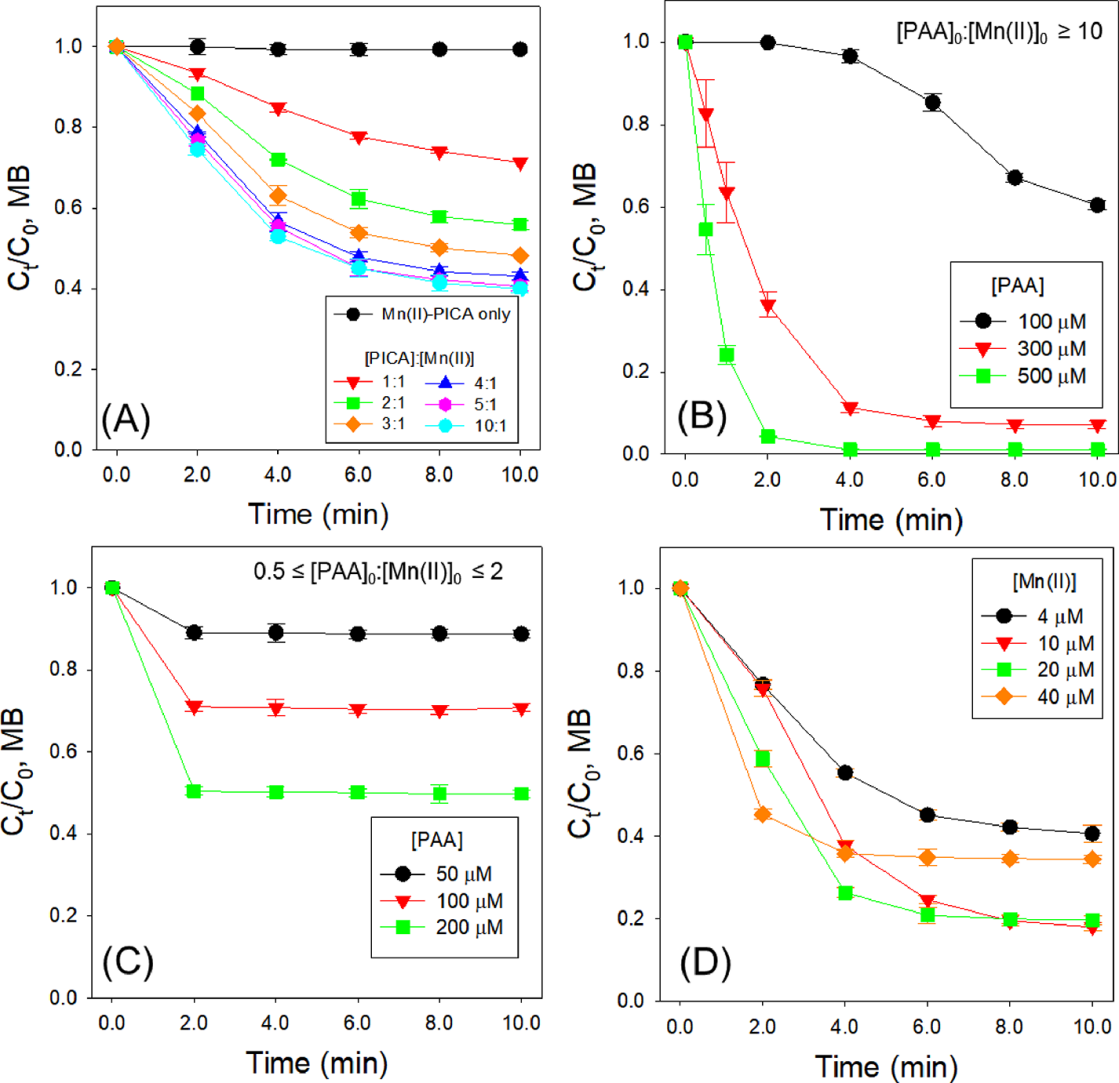
Degradation of MB in the PAA-Mn(II)-PICA system under different reaction conditions. (A) Effects of the molar ratio of Mn(II) to PICA. (B–D) Effects of the molar ratio of Mn(II) to PAA (conditions: [MB]_0_ = 15 μM, *T* = 22 ± 1 °C, (A) [PAA]_0_ = 200 μM, [Mn(II)]_0_ = 4.0 μM, [PICA]_0_ = 4.0–40 μM, initial pH = 5.0; (B) [PAA]_0_ = 100–500 μM, [Mn(II)]_0_ = 10 μM, [PICA]_0_ = 50 μM, initial pH = 5.0; (C) [PAA]_0_ = 50–200 μM, [Mn(II)]_0_ = 100 μM, [PICA]_0_ = 500 μM, initial pH = 5.0; (D) [PAA]_0_ = 200 μM, [Mn(II)]_0_ = 4.0–40 μM, [PICA]_0_ = 20–200 μM, [Mn(II)]:[PICA] ratio = 1:5, initial pH = 5.0).

First, the PAA-Mn(II) system without PICA showed almost no removal of MPs (<0.7%) (Figure 2A). The degradation % of MPs ([MP]_removal,%_) and the initial first-order rate constant (*k*_initial_ in min^–1^) for the MP degradation were used to compare the efficiency of MP abatement. [MP]_removal,%_ was obtained for a reaction time of 10 min. *k*_initial_ in min^–1^ was calculated by the slope of ln(*C*_t_/*C*_0_) versus time at the initial stage of the reaction, where the reaction could be considered to follow pseudo-first-order kinetics (Figure S3).

#### Effect of Mn(II) to PICA Molar Ratio

The effect of PICA on the degradation of a model compound, MB, by the PAA-Mn(II) system was investigated. The addition of PICA significantly enhanced MB degradation by PAA-Mn(II) (Figure 2A and Table S2). Different molar ratios of [Mn(II)]:[PICA] ranging from 1:1 to 1:10 were investigated (conditions: [MB]_0_ = 15 μM, [PAA]_0_ = 200 μM, [Mn(II)]_0_ = 4.0 μM, [PICA]_0_ = 4.0–40 μM, initial pH = 5.0). An increase of the [Mn(II)]:[PICA] ratio from 1:1 to 1:4 led to an increase in both *k*_initial_ and [MB]_removal,%_ (*k*_initial_ from (4.10 ± 0.12) × 10^–2^ to (1.28 ± 0.05) × 10^–1^ min^–1^, [MB]_removal,%_ from 29% to 58%). However, above the [Mn(II)]:[PICA] ratio of 1:4, [MB]_removal,%_ increased only slightly (from 58% to 60%) and the *k*_initial_ value plateaued (Figure S4A).

Ligand exchange kinetics of Mn(II) are rapid; thus, the complexation reactions of Mn(II) with different PICA species are expected to be at equilibrium in the reaction solutions. Five monomeric species are likely available: free Mn ion (Mn^2+^), free PICA^–^, monoligated species (Mn(PICA)^+^ (the complex formation constant (log β_1_) = 4.00), bis-ligated species (Mn(PICA)_2_ (log β_2_ = 7.10), and tris-ligated species (Mn(PICA)_3_^–^ (log β_2_ = 8.80)).^52^ Concentrations of Mn(II) species were estimated with known complex formation constants (Table S3). As shown in Table S3, the concentration of complexed Mn(II) species ([Mn(II)-PICA]_T_ = [Mn(PICA)^+^] + [Mn(PICA)_2_] + [Mn(PICA)_3_^–^]) linearly increases from 4.53 × 10^–2^ to 4.17 × 10^–1^ μM with respect to the [Mn(II)]:[PICA] ratio. While the formation of the Mn(II)-PICA complex linearly increases, MB degradation was not improved when the [Mn(II)]:[PICA] ratio exceeded 1:4. These results indicated that high concentrations of PICA, above the optimal ratio reflecting the overall effect of the Mn(II)-PICA complex and possible scavenging of reactive species by PICA, do not benefit MP degradation.

In this study, the [Mn(II)]:[PICA] ratio of 1:5 was selected to further assess the impacts of different reaction conditions (i.e., PAA and Mn(II) dosages, solution pH, coexistent H_2_O_2_ and acetic acid, water matrix constituents).

#### Effects of PAA and Mn(II) Dosages

The effects of PAA and Mn(II) dosages on MB degradation by the PAA-Mn(II)-PICA system were investigated. First, the effect of PAA dosage was investigated at the initial pH of 5.0 under two reaction conditions (molar ratio [PAA]_0_:[Mn(II)]_0_ ≥ 10 and 0.5 ≤ [PAA]_0_:[Mn(II)]_0_ ≤ 2). When [PAA]_0_ was more than 10 times greater than [Mn(II)]_0_ (excess PAA; conditions: [MB]_0_ = 15 μM, [PAA]_0_ = 100, 300, and 500 μM, [Mn(II)]_0_ = 10 μM, [PICA]_0_ = 50 μM; Figure 2B), MB degradation continued throughout the reaction time course. In spite of [Mn(II)]_0_ < [MB]_0_, MB was completely removed within 4 min at [PAA]_0_:[Mn(II)]_0_ = 50. The *k*_initial_ value increased linearly from (1.95 ± 0.55) × 10^–2^ to (1.21 ± 0.09) × 10^0^ min^–1^ with increasing PAA concentration from 100 to 500 μM (Table S2 and Figure S4B). These results indicated that excess PAA could benefit MP degradation by allowing continuous reactions between PAA and Mn species, resulting in more reactive species. In contrast, when [PAA]_0_ was similar to [Mn(II)]_0_ (conditions: [MB]_0_ = 15 μM, [PAA]_0_ = 50, 100, and 200 μM, [Mn(II)]_0_ = 100 μM, [PICA]_0_ = 500 μM; Figure 2C), MB degradation did not occur after 2 min, which is likely due to PAA depletion. Despite the presence of sufficiently high concentrations of Mn(II) and PAA in comparison to MB, [MB]_removal,%_ was only 11–50%. This indicated that reactive species might be scavenged, likely by excess PICA. Note that, at 0.5 ≤ [PAA]_0_:[Mn(II)]_0_ ≤ 2, the concentration of the uncomplexed PICA is 4.33 × 10^2^ μM, about 9 times greater than that at [PAA]_0_:[Mn(II)]_0_ ≥ 10 (4.87 × 10^1^ μM) (Table S4).

The effect of Mn(II) dosage (4.0–40 μM) was investigated at the initial pH of 5.0 while fixing [PAA]_0_ at 200 μM and the [Mn(II)]:[PICA] ratio of 1:5 (Figure 2D). The *k*_initial_ value increased from (1.37 ± 0.04) × 10^–1^ to (2.82 ± 0.19) × 10^–1^ min^–1^ when the Mn(II) concentration was increased from 4.0 to 20 μM; however, the increase to 40 μM of the Mn(II) concentration decreased the *k*_initial_ value to (2.14 ± 0.36) × 10^–1^ min^–1^ (Table S2 and Figure S4C), indicating that reactive species could be scavenged by excess Mn(II) and PICA.

#### Effect of Initial pH

**Figure 3:**
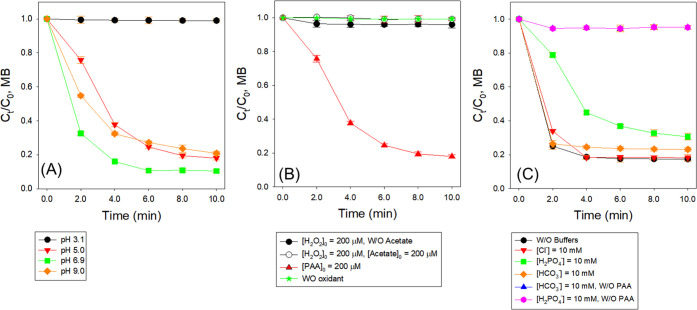
Degradation of MB in the PAA-Mn(II)-PICA system under different reaction conditions. (A) Effects of initial pH. (B) Effects of coexistent H_2_O_2_ and acetic acid. (C) Effects of buffer anions (conditions: [MB]_0_ = 15 μM, *T* = 22 ± 1 °C, (A) [PAA]_0_ = 200 μM, [Mn(II)]_0_ = 10 μM, [PICA]_0_ = 50 μM, initial pH = 3.1–9.0; (B) [PAA or H_2_O_2_]_0_ = 200 μM, [acetate]_0_ = 0 or 200 μM, [Mn(II)]_0_ = 10 μM, [PICA]_0_ = 50 μM, initial pH = 5.0; (C) [PAA]_0_ = 200 μM, [Mn(II)]_0_ = 20 μM, [PICA]_0_ = 100 μM, [Cl^–^ or HCO_3_^–^ or H_2_PO_4_^–^/HPO_4_^2–^]_0_ = 10 mM, initial pH = 7.1).

The degradation of MB by the PAA-Mn(II)-PICA system was investigated in the initial pH range of 3.1–9.0 (Figure 3A, conditions: [MB]_0_ = 15 μM, [PAA]_0_ = 200 μM, [Mn(II)]_0_ = 10 μM, [PICA]_0_ = 50 μM). Note that solution pH decreased by 0–2.6 units during the reaction, which is likely due to the formation of highly Lewis acidic high-valent Mn species.^53^ The *k*_initial_ values of MB degradation by the PAA-Mn(II)-PICA system were (1.64 ± 0.02) × 10^–3^, (2.30 ± 0.15) × 10^–1^, (4.10 ± 0.32) × 10^–1^, and (2.41 ± 0.20) × 10^–1^ min^–1^ at pH 3.1, pH 5.0, pH 6.9, and pH 9.0, respectively, and were in the order of pH 6.9 > pH 9.0 > pH 5.1 ≫ pH 3.1 (Table S5 and Figure S4D). The effect of initial pH on MB degradation by the PAA-Mn(II)-PICA system was likely attributed to the change of the speciation of PICA and PAA, while MB speciation had little change within the pH range (p*K*_a_ of MB < 1.0). PICA has a p*K*_a_ value of 5.39 for pyridinium N (structure shown in Table S1).^54^ In general, a deprotonated ligand will be a stronger σ-donor;^55^ thus, PICA could form a stronger complex with Mn(II) at higher pH. Additionally, protonation of pyridinium nitrogen eliminates the ligand’s ability to chelate metal and causes PICA to coordinate only through carboxylate oxygen. Some combination of these effects is the most plausible explanation for little degradation of MB at pH 3.1. PAA has a p*K*_a_ value of 8.2,^56^ and the deprotonated PAA (PAA^–^) is a weaker oxidant than neutral PAA (PAA^0^). In the pH range of 3.1–6.9, PAA^0^ is predominant (fraction of PAA^0^ (*f*_PAA_^0^) = 1.00–0.95), and the *f*_PAA_^0^ decreases to 0.14 at pH 9.0.

#### Effects of Coexistent H_2_O_2_ and Acetic Acid

As previously noted, H_2_O_2_ and acetic acid coexist in the PAA solution; thus, it is worth mentioning that the degradation of MB by H_2_O_2_-Mn(II)-PICA and H_2_O_2_-acetic acid-Mn(III)-PICA was minimal (Figure 3B, conditions: [MB]_0_ = 15 μM, [H_2_O_2_]_0_ = 200 μM, [acetic acid] = 0 or 200 μM, [Mn(II)]_0_ = 10 μM, [PICA]_0_ = 50 μM, initial pH = 5.0). Moreover, the presence of additional H_2_O_2_ did not influence the degradation of MB by Mn(II)-PICA (conditions: [MB]_0_ = 15 μM, [PAA]_0_ = 200 μM, [H_2_O_2_]_0_ = 184 μM (84 μM in PAA solution and additional input of 100 μM), [Mn(II)]_0_ = 20 μM, [PICA]_0_ = 100 μM, initial pH = 5.0; Figure S5). These findings indicated that H_2_O_2_ and acetic acid coexistent in PAA solution played a minimal role in degrading MPs in the PAA-Mn(II)-PICA system.

#### Effects of Water Matrix Constituents

The impacts of water matrix constituents (i.e., chloride (Cl^–^), bicarbonate (HCO_3_^–^), and phosphate (H_2_PO_4_^–^/HPO_4_^2–^)) on MB degradation by the PAA-Mn(II)-PICA system was investigated at the initial pH of 7.1 (Figure 3C and Table S6, conditions: [MB]_0_ = 15 μM, [PAA]_0_ = 200 μM, [Mn(II)]_0_ = 20 μM, [PICA]_0_ = 100 μM, [Cl^–^ or HCO_3_^–^ or H_2_PO_4_^–^/HPO_4_^2–^]_0_ = 2.0–10 mM, initial pH = 7.1). Note that MB was minimally degraded by PAA-Cl^–^, PAA-HCO_3_^–^, PAA-H_2_PO_4_^–^/HPO_4_^2–^, Mn(II)-PICA-Cl^–^, Mn(II)-PICA-HCO_3_^–^, and Mn(II)-PICA-H_2_PO_4_^–^/HPO_4_^2–^ (Figure S6A).

The presence of Cl^–^ did not impact MB degradation by the PAA-Mn(II)-PICA system. Note that PAA systems with other metal ions (i.e., Co(II), Fe(III)-PICA, and Ru(III)) also showed minimal influence of Cl^–^.^24,26,29,42^ The presence of HCO_3_^–^ had minimal impact on the MB degradation by PAA-Mn(II)-PICA (Figure S6B). In contrast, previous research reported minimal to moderate impacts of HCO_3_^–^ on PAA-Ru(III) and PAA-Fe(III)-PICA,^26,42^ while having a significant inhibitory effect on PAA-Co(II).^24,29^

Compared to Cl^–^ and HCO_3_^–^, H_2_PO_4_^–^/HPO_4_^2–^ moderately retarded MB degradation by the PAA-Mn(II)-PICA system (*k*_initial_ decreasing from (2.78 ± 0.24) × 10^–1^ to (2.14 ± 0.35) × 10^–1^ min^–1^) and reduced the overall abatement from 80.5% to 68.9% (Figure S6C). The complex formation constant of Mn(II) anion is 0.6 for Mn(II)-Cl^–^, 4.7 for Mn(II)-CO_3_^2–^, 11.6 for Mn(II)-HCO_3_^–^, and 15.8 for Mn(II)-HPO_4_^2–^.^57^ When the anion concentration is 10 mM, Mn(II) forms only weak complexes with Cl^–^ (*f*_MnCl_ = 0.01) but forms strong complexes with HCO_3_^–^ () and H_2_PO_4_^–^/HPO_4_^2–^ (*f*_MnHPO_4__ = 0.83). Despite the strong complexation of Mn(II) with HCO_3_^–^ and H_2_PO_4_^–^/HPO_4_^2–^, MB degradation was only slightly to moderately impeded, indicating that PICA could still competitively interact with Mn(II) (and intermediate Mn species) and mediate the reaction. The above results suggest that environmental waters containing high concentrations of H_2_PO_4_^–^/HPO_4_^2–^ will exert some inhibitory effect on MB degradation by the PAA-Mn(II)-PICA system through complexation competition.

### Major Reactive Species in the PAA-Mn(II)-PICA System

#### Impacts of Mn(III)

**Figure 4:**
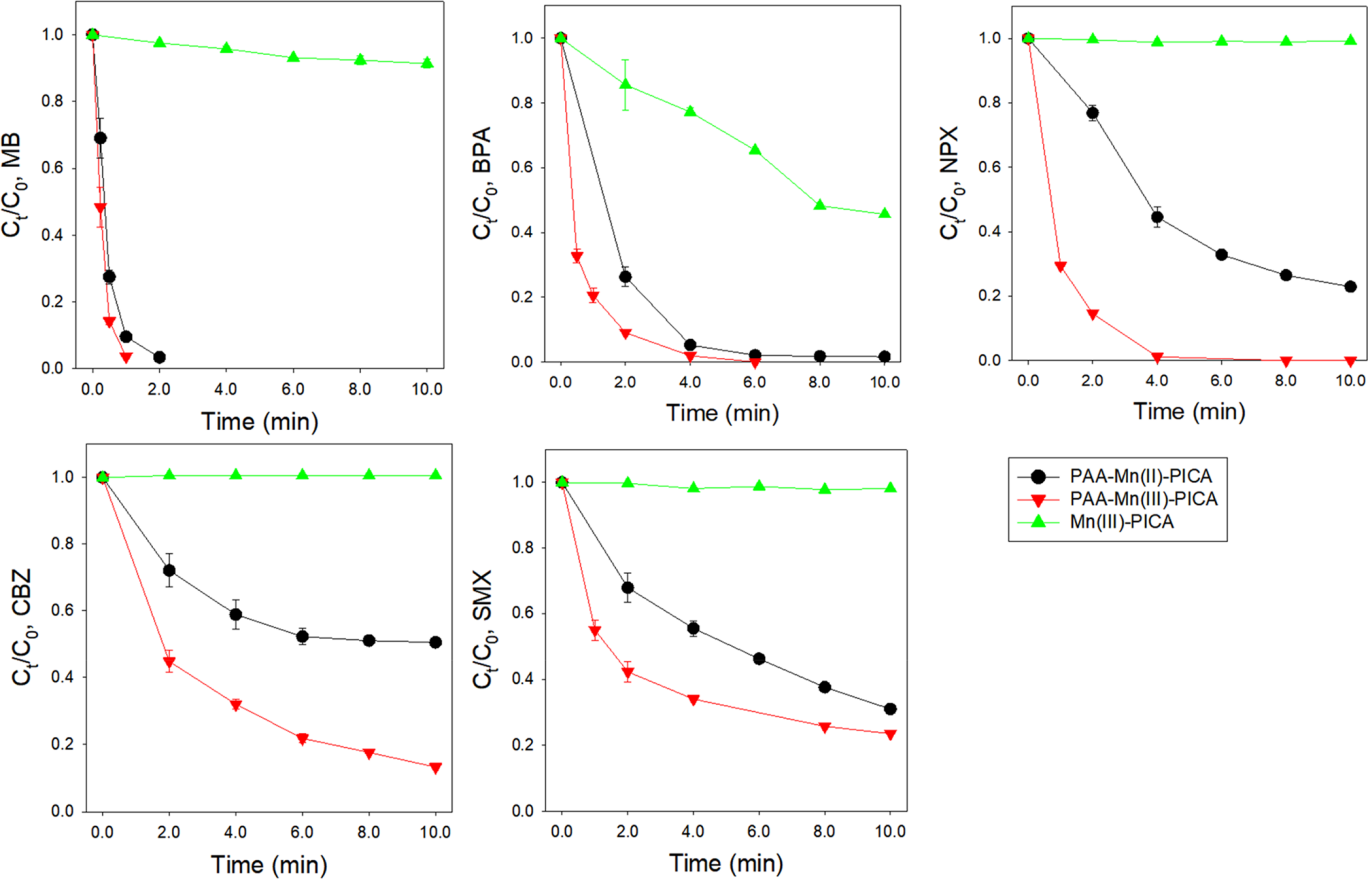
Degradation of MPs (MB, BPA, NPX, CBZ, and SMX) by the Mn(III)-PAA, PAA-Mn(II)-PICA, and PAA-Mn(III)-PICA systems. (conditions: [MP]_0_ = 15 μM, [PAA]_0_ = 0 or 500 μM, [Mn(II) or Mn(III)]_0_ = 20 μM [PICA]_0_ = 100 μM, initial pH = 7.0, *T* = 22 ± 1 °C).

Mn(III) formed by the reaction of Mn(II) with PAA could be an oxidant to oxidize MPs. Mn(III) is unstable and rapidly disproportionates to Mn(II) and Mn(IV).^58^ It has been demonstrated that the presence of ligands, such as pyrophosphate, ethylenediaminetetraacetic acid (EDTA), and nitrilotrismethylenephosphonic acid (NTA), stabilizes Mn(III) at neutral pH.^59−61^ Similarly, in this study, the presence of PICA stabilized Mn(III), thus generating minimal Mn(IV) (colloidal MnO_2_), which was confirmed by no peak observed at 350–420 nm in Mn(III)-PICA solution (Figure S7B). Separately, we investigated the impact of Mn(III)-PICA on the degradation of MPs (MB, BPA, CBZ, NPX, and SMX). Degradation of CBZ, NPX, and SMX was negligible during 10 min, but MB and BPA were degraded to some extent with [MP]_removal,%_ of 8.6% and 54.4%, respectively (Figure 4; conditions: [MP]_0_ = 15 μM, [Mn(III)]_0_ = 20 μM, [PICA]_0_ = 100 μM, initial pH = 7.0). The reactivities of soluble Mn(III) toward MPs are scarcely reported, but several studies reported the selectivity of Mn(III) in degrading MPs. Jiang et al.^62^ reported that Mn(III) did not show reactivity toward CBZ, while Mn(III) was an intermediate enhancing the degradation of BPA during Mn(VII) oxidation in another study.^63^ Sun et al.^64^ reported enhanced degradation of MB by Mn(III) formed by the activation of MnO_2_ by S(IV). Our results generally corroborate with the previous studies.

Next, the degradation of MPs by PAA-Mn(III)-PICA was compared to that in the PAA-Mn(II)-PICA system (Figure 4 and Table S7). Significant degradation of MPs was also observed in the PAA-Mn(III)-PICA system during 10 min. The *k*_initial_ values were greater than those in the PAA-Mn(II)-PICA system ((0.18–3.42) × 10^0^ > (0.12–2.36) × 10^0^ min^–1^ for PAA-Mn(II)-PICA and PAA-Mn(II)-PICA systems, respectively). It is worth comparing between Mn(III)-PICA and PAA-Mn(III)-PICA for MP degradation. The addition of PAA accelerated MP degradation, and the *k*_initial_ values increased from (0–8.10) × 10^–2^ to (0.18–3.42) × 10^0^ min^–1^.

#### Formation of High-Valent Mn Species

**Figure 5:**
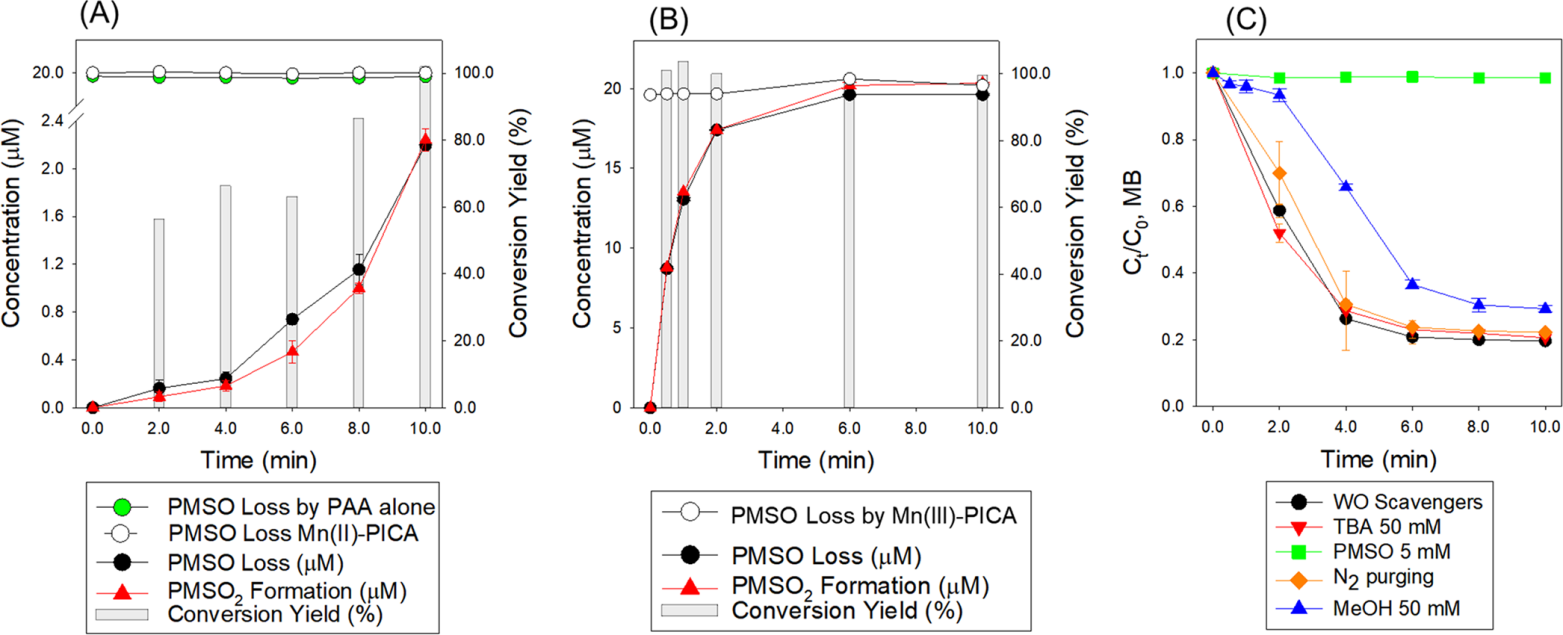
(A, B) PMSO loss ([PMSO]_0_–[PMSO]_t_) and PMSO_2_ formation ([PMSO_2_]_t_) by the PAA-Mn(II)-PICA and PAA-Mn(III)-PICA oxidation process. Green circle dots show the change of PMSO concentration ([PMSO]_t_) by PAA alone (A). Empty dots show the change of PMSO concentration ([PMSO]_t_) by Mn(II)-PICA and Mn(III)-PICA alone. (C) Degradation of MB by PAA-Mn(II)-PICA in the presence and absence of scavengers (conditions: (A and B) [PMSO]_0_ = 20 μM, [PAA]_0_ = 200 μM, [Mn(II) or Mn(III)]_0_ = 20 μM, [PICA]_0_ = 100 μM, initial pH = 5.0, *T* = 22 ± 1 °C; (C) [MB]_0_ = 15 μM, [PAA]_0_ = 200 μM, [Mn(II)]_0_ = 20 μM, [PICA]_0_ = 100 μM, [TBA]_0_ = 0 or 50 mM, [MeOH]_0_ = 0 or 50 mM, [PMSO]_0_ = 0 or 5.0 mM, initial pH = 5.0, *T* = 22 ± 1 °C).

High-valent Mn species (Mn(IV/V) (from eqs 2–4) are possible reactive species that could be formed in the PAA-Mn(II)-PICA system. First, we confirmed that Mn(IV) was not formed during the reaction (no peak at 350–420 nm, Figure S7C,D). Then, the probe compound, PMSO, was used to distinguish high-valent Mn species (V) and free radicals in PAA-Mn(II)-PICA and PAA-Mn(III)-PICA systems (conditions: [PAA]_0_ = 200 μM, [Mn(II) or Mn(III)]_0_ = 20 μM, [PICA]_0_ = 100 μM, [PMSO]_0_ = 20 μM, initial pH = 5.0, Figure 5A,B). Note that PMSO minimally reacts with PAA. Oxidation by high-valent metal species converts PMSO to PMSO_2_ via oxygen atom transfer, whereas oxidation by free radicals generates hydroxylated or polymeric products of PMSO.^65^ Additionally, CH_3_C(O)O^•^/CH_3_C(O)OO^•^ minimally reacts with PMSO.^66^ In both PAA-Mn(II)-PICA and PAA-Mn(III)-PICA systems, PMSO was converted to PMSO_2_ over time with high conversion yield (∼100% in 10 min), suggesting high-valent Mn species were the major reactive species, rather than free radicals. Note that control experiments confirmed that PMSO was not degraded by PAA alone, Mn(II)-PICA, or Mn(III)-PICA. The conversion of PMSO to PMSO_2_ in the PAA-Mn(III)-PICA system was faster than that in the PAA-Mn(II)-PICA system, which was likely due to (i) Mn(III)-PICA reacting with PAA faster than Mn(II)-PICA owing to higher reactivity and/or (ii) the reaction of Mn(II)-PICA with PAA initially forming Mn(III) which was further converted to high-valent Mn species (Mn(V)). Further oxidized species (Mn(VII) could be ruled out, because it was confirmed that Mn(VII) was not formed during the reaction (no peak at 525 nm, Figure S7C,D). Additionally, previous studies have reported that Mn(VI/VII) degrades PMSO much more slowly than Mn(V).^67,68^

#### Contribution of Reactive Species to MP Degradation

The contribution of reactive species to MB degradation was evaluated by adding several scavengers (Figure 5C, conditions: [MB]_0_ = 15 μM, [PAA]_0_ = 200 μM, [Mn(II)]_0_ = 20 μM, [PICA]_0_ = 100 μM, initial pH = 5.0). Addition of 50 mM TBA minimally influenced the degradation of MB, indicating that ^•^OH was not important. MeOH is also a well-known scavenger for free radicals including ^•^OH (*k*_•OH/TBA_ = 6.0 × 10^8^ M^–1^·s^–1^).^69^ Meanwhile, MeOH is a probable scavenger for CH_3_C(O)O^•^/CH_3_C(O)OO^•^, as Wang et al.^23^ reported that MeOH suppressed the degradation of MP in the PAA-Co(II) system where little ^•^OH is formed while CH_3_C(O)O^•^/CH_3_C(O)OO^•^ plays a major role in MP degradation. Note that Mn(IV) is inert to MeOH, but Mn(V) may have some reactivity with MeOH.^70^ Adding 50 mM MeOH slightly retarded MB degradation (*k*_initial_ was decreased from (2.82 ± 0.19) to (2.13 ± 0.20) × 10^0^ min^–1^, Table S8), suggesting that CH_3_C(O)O^•^/CH_3_C(O)OO^•^ and/or Mn(V) could contribute to MB degradation.

Then, the relative contributions of CH_3_C(O)O^•^/CH_3_C(O)OO^•^ versus Mn(V) to MB degradation by PAA-Mn(II)-PAA was assessed by using PMSO. Addition of 5.0 mM PMSO completely inhibited the degradation of MB, indicating the importance of high-valent Mn species (Mn(V)) in MB degradation in the PAA-Mn(II)-PICA system, and the contribution of CH_3_C(O)O^•^/CH_3_C(O)OO^•^ was minimal. The impact of O_2_ was also assessed by investigating the degradation in a purged reaction solution, and MB degradation by the PAA-Mn(II)-PICA system was minimally influenced by the presence or absence of O_2_. Overall, with limited impact from TBA but significant inhibition of MB degradation by PMSO as well as a high conversion yield of PMSO to PMSO_2_, high-valent Mn species (Mn(V)), rather than radicals, were likely the predominant reactive species leading to MPs’ degradation.

#### Other Minor Reactive Species

First, as mentioned above, we confirmed that MPs’ degradation in H_2_O_2_-Mn(II)-PICA was limited (Figure 3B), indicating that the reactive species generated from the reaction of H_2_O_2_ with Mn (including high-valent species)-PICA played a minor role in MPs’ degradation. Minimal impact of TBA ruled out the contribution of ^•^OH to MPs’ degradation (Figure 5C). HO_2_^•^/O_2_^•–^ has low reactivity with MPs (*k*_HO_2_^•^__/MPs_ ≈ 0–10^8^ M^–1^·s^–1^).^71^ Other radicals, such as ^•^CH_3_, CH_3_OO^•^, and CH_3_(O)O^•^ could be generated by the secondary reactions.^42^ However, those radicals have minimal impact on MPs’ degradation because of low reactivity with MPs (*k*_CH_3_OO^•^__/MPs_ ≈ 10^5^–10^7^ M^–1^·s^–1^),^72^ the rapid reaction with O_2_ (*k*_^•^CH_3_/O_2__ = 4.1 × 10^9^ M^–1^ s^–1^),^73^ and rapid self-decay (*k*_CH_3_C(O)O^•^_ ∼ 1 × 10^5^ s^–1^).^32^

### Application of the PAA-Mn(II)-PICA Oxidation Process for the Removal of MPs

**Figure 6:**
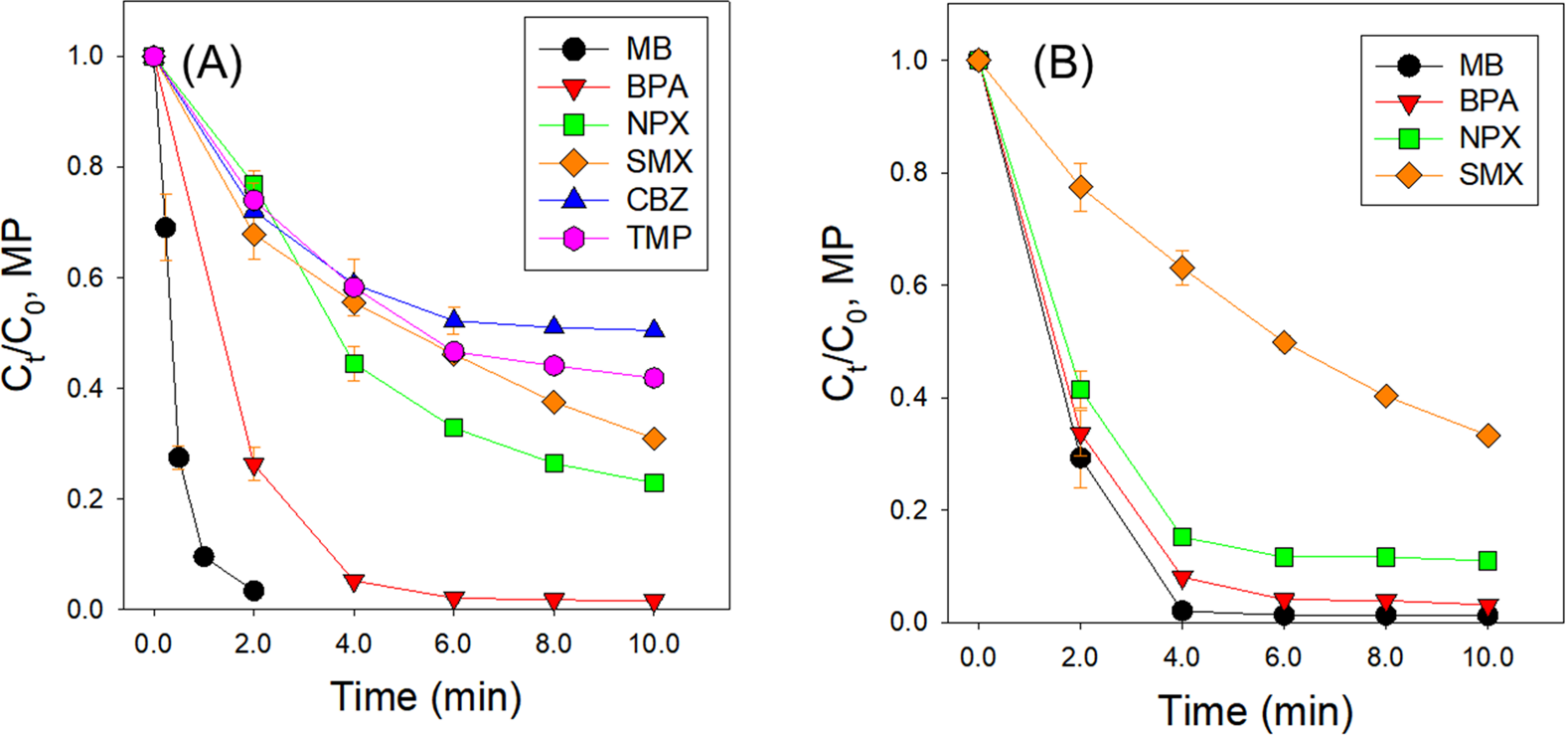
Degradation of MPs in reagent water (A) and tertiary effluent from a wastewater treatment plant (B) by the PAA-Mn(II)-PICA oxidation process (conditions: [MP]_0_ = 15 μM, [PAA]_0_ = 500 μM, [Mn(II)]_0_ = 20 μM, [PICA]_0_ = 100 μM, initial pH = 7.0 for (A), pH 6.5 for (B), *T* = 25 ± 1 °C). MPs: MB, methylene blue; BPA, bisphenol A; NPX, naproxen; SMX, sulfamethoxazole; CBZ, carbamazepine; TMP, trimethoprim.

The removal of an additional five MPs (NPX, CBZ, BPA, TMP, and SMX) by the PAA-Mn(II)-PICA system was investigated at pH 7.0 (conditions: [MPs]_0_ = 15 μM, [PAA]_0_ = 500 μM, [Mn(II)]_0_ = 20 μM, [PICA]_0_ = 100 μM, initial pH = 7.0, Figure 6A). More than 50% removal of MPs was achieved in 10 min. When compared to *k*_initial_ values and [MP]_removal,%_ at 10 min, the degradation efficiency was in the order of MB > BPA > NPX > SMX > TMP > CBZ. There are few available rate constants for the reaction of high-valent Mn species with MPs, but it has been demonstrated that Mn(V) has high reactivities with a range of MPs containing electron-rich moieties such as phenolic, olefin, and sulfoxide.^74^ High reactivity of Mn(V) toward phenolic compounds could be due to the ability to generate O-bridged compounds, allowing inner-sphere electron transfer.^70,75^ It also has been demonstrated that Mn(V) could oxidize sulfoxide and olefin moieties via oxygen-atom transfer or alkene addition reaction.^76,77^ The results in this study also showed rapid degradation of BPA containing phenolic moiety and SMX and MB with sulfoxide moiety.

The feasibility of the PAA-Mn(II)-PICA system to degrade MPs (MB, NPX, SMX, and BPA) in real water matrices was assessed in a tertiary wastewater effluent. Results showed minimal to mild impact of real water matrices, and >66% of MPs’ degradation was achieved (Figure 6B). These results demonstrate the effectiveness of the PAA-Mn(II)-PICA system for degrading MPs in wastewater containing complex matrix components such as organic matter and divalent metals.

### Comparison to the PAA-Fe(III)-PICA System

The chelating agent PICA significantly enhances the capability of both Mn(II) and Fe(III) to activate PAA for MP degradation.^42^ Both PAA-Mn(II)-PICA and PAA-Fe(III)-PICA systems showed much less degradation of MPs at acidic pH than at higher pH, due to protonation of PICA hindering metal complexation. Under comparable reaction conditions (i.e., 1:5 metal-to-PICA molar ratio and PAA dose), both systems could achieve a high percentage of MP degradation at the initial pH of 7 within 10 min (>56% for Fe(III): [PAA]_0_ = 500 μM, [Fe(III)]_0_ = 50 μM, [PICA]_0_ = 125 μM; >50% for Mn(II): [PAA]_0_ = 200 μM, [Mn(II)]_0_ = 20 μM, [PICA]_0_ = 100 μM). The presence of Cl^–^ has little impact on MP degradation in both systems. In the PAA-Fe(III)-PICA system, HCO_3_^–^ moderately reduces MP abatement from 90% to 77%, while H_2_PO_4_^–^/HPO_4_^2–^ completely inhibits MP degradation. Comparatively, the inhibitory effects of HCO_3_^–^ and H_2_PO_4_^–^/HPO_4_^2–^ are weaker in the PAA-Mn(II)-PICA system. Different effects of anions are likely due to their different ability to compete with PICA for complexing Fe(III) versus Mn(II). High-valent metal species, rather than radicals, are major reactive species contributing to MP degradation in both systems. PAA shows little reactivity for several transition metals, such as Fe(III), Mn(II), Mn(III), Cu(II), and Ni(II).^27^ It would be worthwhile to expand research further into the application of PICA to such transition metals in PAA systems.

### Environmental Significance and Implications

The PAA-Mn(II) AOP requires high dosages of PAA and Mn(II) to achieve sufficient efficiency for MP removal. This study showed that the chelating agent, PICA, can dramatically enhance the efficiency of PAA-Mn(II) AOP to degrade a range of MPs in the pH range of 3.0–9.0. With low dosages of PAA and Mn(II), significant abatement of MPs was achieved, and the impacts of real water matrices were minimal, demonstrating the PAA-Mn(II)-PICA system to be a promising AOP. The robust evaluation suggested that high-valent Mn species (Mn(V)) were likely the major reactive species contributing to MP degradation by the PAA-Mn(II)-PICA system. The minimal impacts of water matrices could be attributed to the formation of selective Mn(V). A variety of chelating agents, such as EDTA and NTA, have been applied to metal ion-based AOPs to improve the efficiency of catalytic activity for oxidants. PICA is less toxic and more biodegradable than other chelating agents, which is beneficial in minimizing negative environmental risks.^45,78^ Furthermore, among metals, Mn has low toxicity and high abundance in natural environments, while PAA exhibits several advantages over other conventional oxidants. Thus, the PAA-Mn(II)-PICA process could be a promising AOP that is suitable for a wide pH range and complex water matrices. Further research is needed to evaluate in detail the potential impacts of degradation products of MPs as well as PICA and its products.

## Supporting Information Available

Chemicals, reagents, and analytical methods (Text S1); chemical properties of micropollutants and probe compounds (Table S1); *k*_initial_ of degradation of MB by PAA-Mn(II)-PICA under different reaction conditions (Tables S2, S5, S6, and S8); species of Mn(II) (Table S3 and S4); *k*_initial_ of degradation of MPs (MB, BPA, NPX, CBZ, SMX, and TMP) by PAA-Mn(II)-PICA and/or PAA-Mn(III)-PICA (Table S7); continuous PAA decrease by Mn(II)-PICA (Figure S1); PAA decrease by Mn(II)-PICA with and without additional H_2_O_2_ input (Figure S2); initial reaction kinetics of MB by PAA-Mn(II)-PICA (Figure S3); *k*_initial_ of degradation of MB by PAA-Mn(II)-PICA (Figure S4); MB degradation by Mn(II)-PICA with and without additional H_2_O_2_ input (Figure S5); effects of various anions (Figure S6); UV–vis spectra of Mn(III) and Mn(III)-PICA, PAA-Mn(II)-PICA, and PAA-Mn(III)-PICA (Figure S7) (PDF)
