# Supplementary material for: Picolinic Acid-Mediated Catalysis of Mn(II) for Peracetic Acid Oxidation Processes: Formation of High-Valent Mn Species
Source: Environ Sci Technol. 2023 May 24;57(47):18929–39. doi: 10.1021/acs.est.3c00765 (PMC10690714; doi:10.1021/acs.est.3c00765)
Supplement: Supplementary file 1 — es3c00765_si_001.pdf [file es3c00765_si_001.pdf]

Supporting Information

for

Picolinic Acid-Mediated Catalysis of Mn(II) for Peracetic Acid Oxidation Processes: Formation of High Valent Mn Species

Juhee Kim,[a] Junyue Wang,[a] Daniel C. Ashley,[b] Virender K. Sharma,[c,*] Ching-Hua Huang[a,*]

[a]School of Civil and Environmental Engineering,
Georgia Institute of Technology, Atlanta, GA 30332, United States

[b]Department of Chemistry and Biochemistry, Spelman College, Atlanta, GA 30314, United States

[c]Department of Environmental and Occupational Health, School of Public Health, Texas A&M University, College Station, Texas 77843, United States

[*]Corresponding Authors. Emails: ching-hua.huang@ce.gatech.edu (Ching-Hua Huang), vsharma@tamu.edu (Virender K. Sharma)

Number of Pages: 15

Number of Texts: 1

Number of Tables: 8

Number of Figures: 7

Contents

Texts

Text S1. Information on chemicals, reagents and analytical methods. ---------------------------S3

Tables

Table S1. Chemical properties of picolinic acid, micropollutants and probe compounds.-----S5

Table S2. Initial first-order rate constants (k_{initial}) of degradation of MB by PAA-Mn(II)-PICA of [PAA]:[Mn(II)]:[PICA].--- S6

Table S3. Species of Mn(II) (μM and the percentage) in Figure 2A.--------------------------- S6

Table S4. Species of Mn(II) (μM and the percentage) in Figure 2B and 2C.------------------- S6

Table S5. Initial first-order rate constants (k_{initial}) of degradation of MB by PAA-Mn(II)-PICA at initial pH 3.1–9.0.--- S7

Table S6. Initial first-order rate constants (k_{initial}) of degradation of MB by PAA-Mn(II)-PICA in the presence of various anions.--- S7

Table S7. Initial first-order rate constants (k_{initial}) of degradation of MPs (MB, BPA, NPX, CBZ, SMX and TMP) by PAA-Mn(II)-PICA and/or PAA-Mn(III)-PICA.---------------- S8

Table S8. Initial first-order rate constants (k_{initial}) of degradation of MB by PAA-Mn(II)-PICA in the presence of scavengers.--- S8

Figures

Figure S1. continuous PAA decrease by Mn(II)-PICA. ---S9

Figure S2. PAA decrease by Mn(II)-PICA with and without additional H_2O_2 input. ---------S10

Figure S3. Initial reaction kinetics of MB by PAA-Mn(II)-PICA with (A) different molar ratio of Mn(II) to PICA (data is from Figure 2A) and (B) different pHs (data is from Figure 3A): $\ln(C_t/C_0)$ versus time relationships. -------------------------------------S11

Figure S4. Initial first-order rate constants (k_{initial}) of degradation of MB by PAA-Mn(II)-PICA with (A) different ratio of [PICA]:[Mn(II)], (B) $[PAA]_0$, (C) $[Mn(II)]_0$ and (D) initial pH. --S12

Figure S5. MB degradation by Mn(II)-PICA with and without additional H_2O_2 input. ------S13

Figure S6. Effects of various anions on MB degradation. --------------------------------------S14

Figure S7. (A) Photographs of Mn(III) and Mn(III)-PICA solution (pH 5, Mn(III):[PICA]=1:0 or 1:5). UV-vis spectra of (B) Mn(III) and Mn(III)-PICA, (C) PAA-Mn(II)-PICA, and (D) PAA-Mn(III)-PICA.---S15

Text S1. Chemicals and reagents

Peracetic acid (PAA) solution (36% PAA and 5% H_2O_2 w/w in acetic acid and water solution), hydrogen peroxide solution (30% H_2O_2 w/w in water), manganese(II) sulfate ($MnSO_4$), manganese(III) acetate dihydrate ($(CH_3COO)_3Mn{\cdot}2H_2O$) and picolinic acid (PICA) were purchased from Sigma-Aldrich (St. Louis, MO). Target compounds (methylene blue (MB), sulfamethoxazole (SMX), naproxen (NPX), carbamazepine (CBZ), bisphenol-A (BPA), trimethoprim (TMP)), *tert*-butyl alcohol (TBA), methyl phenyl sulfoxide (PMSO), methyl phenyl sulfone ($PMSO_2$) and methanol (MeOH) were purchased from Sigma-Aldrich (St. Louis, MO, USA) or Fisher Scientific (Fair Lawn, NJ, USA) at the highest purity and used without further purification.

Chemicals such as NaOH, H_2SO_4, NaCl, $NaHCO_3$, $Na_2HPO_4{\cdot}7H_2O$, $NaH_2PO_4{\cdot}H_2O$, KI, *N,N*-diethyl-*p*-phenylenediamine (DPD) and others were also purchased from Sigma-Aldrich (St. Louis, MO, USA) or Fisher Scientific (Fair Lawn, NJ, USA) at the highest purity and used without further purification. Reagent-grade deionized (DI) water (>18mΩ-cm) was generated from a Milli-Q nanopure water purification system (Billerica, MA). Working stock solutions of oxidants (100 mM for PAA, 100 mM for H_2O_2), Mn(II) solution (100 mM), PICA solutions (100 mM), and target compounds (1000 μM of MB, and 15 μM of others) were prepared in DI water immediately prior to each set of experiments. The concentrations of PAA and H_2O_2 in the stock solutions were determined by titration as described previously.[1]

The DPD method was used to determine the PAA concentration.[20,47] The MB concentration was determined spectrophotometrically at 665 nm (Beckman DU 520 UV-visible spectrophotometer, Beckman Coulter, Inc., Fullerton, CA, USA). Other MPs (NPX, SMX, BPA, CBZ, TMP), PMSO and $PMSO_2$ were analyzed using an Agilent 1100 high performance liquid chromatography (HPLC)-diode-array detector (DAD) system equipped with an Agilent Zorbax SB–C18 column (2.1 × 150 mm, 5 μm). The compounds were analyzed by using

isocratic mobile phases: 10-90% of acetonitrile and 90-10% of 0.1 % (v/v) formic acid in water at a flow rate of 0.20-0.6 $mL \cdot min^{-1}$, depending on specific compounds. Wavelengths of DAD were set at 230, 268, 230, 268, 257, 230, and 215 nm for NPX, SMX, BPA, CBZ, TMP, PMSO, and $PMSO_2$, respectively.

Table S1. Chemical properties of picolinic acid, micropollutants (MPs) and probe compounds used in this study.

Compound	Category	pK_a (25°C)	log K_{ow}	Water solubility (mg L^{-1}, 25 °C)	Molecular weight (g mol^{-1})	Chemical structure
Picolinic acid (PICA)	-	1.1, 5.39	0.72	9.60×10^{2}	123.11	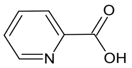
Naproxen (NPX)	Anti-inflammatory	4.2	3.2	1.59×10^{1}	230.26	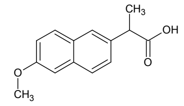
Carbamazepine (CBZ)	Anticonvulsant	13.9	2.4	1.80×10^{1}	236.27	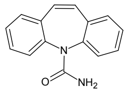
Sulfamethoxazole (SMX)	Antibiotic	1.6, 5.7	0.89	6.10×10^{2} (37°C)	253.28	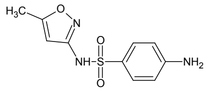
Trimethoprim (TMP)	Antibiotic	7.1	0.91	4.00×10^{2}	290.32	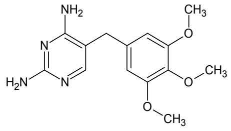
Bisphenol-A (BPA)	Plasticizer	9.2	3.4	1.20×10^{2}	228.29	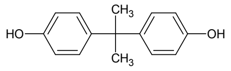
Methylene blue (MB)	Dye	<1.0	2.4	3.60×10^{6}	319.85	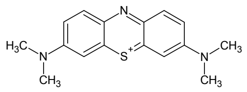
Methyl phenyl sulfoxide (PMSO)	-	-	-	5.06×10^{2}	140.20	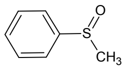

Table S2. Initial first-order rate constants ($k_{initial}$)[a] of degradation of MB by PAA-Mn(II)-PICA at initial pH 5.0 and 22 °C ($R^2 > 0.81$).

Moral ratio (PAA:Mn(II):PICA)	$k_{initial}$ (min^{-1})	Molar ratio (PAA:Mn(II):PICA)	$k_{initial}$ (min^{-1})
**200:4:4**	$(4.10 \pm 0.12)\times10^{-2}$	**200:10:50**	$(2.30 \pm 0.15)\times10^{-1}$
**200:4:8**	$(7.85 \pm 0.03)\times10^{-2}$	**200:20:100**	$(2.82 \pm 0.19)\times10^{-1}$
**200:4:12**	$(1.05 \pm 0.04)\times10^{-1}$	**200:40:200**	$(2.14 \pm 0.36)\times10^{-1}$
**200:4:16**	$(1.28 \pm 0.05)\times10^{-1}$	**100:10:50**	$(1.95 \pm 0.54)\times10^{-2}$
**200:4:20**	$(1.37 \pm 0.04)\times10^{-1}$	**300:10:50**	$(4.90 \pm 0.18)\times10^{-1}$
**200:4:40**	$(1.41 \pm 0.07)\times10^{-1}$	**500:10:50**	$(1.21 \pm 0.09)\times10^{0}$

[a] Rate constants obtained from linear regression of all data points of duplicate experiments and reported with standard error.

Table S3. Species of Mn(II) (μM and the percentage) in Figure 2A.

Total [Mn]	Total [PICA]	$[Mn^{2+}]$	[MnL]	$[MnL_2]$	$[Mn(II)\text{-}PICA]_T$ [a]	Uncomplexed PICA
4	4	3.95 (98.9%)	4.53×10^{-2} (1.1%)	6.52×10^{-5} (0.0%)	4.53×10^{-2} (1.1%)	3.95
4	8	3.91 (97.8%)	8.95×10^{-2} (2.2%)	2.58×10^{-4} (0.0%)	8.98×10^{-2} (2.2%)	7.91
4	12	3.87 (96.7%)	1.33×10^{-1} (3.3%)	5.74×10^{-4} (0.0%)	1.33×10^{-1} (3.3%)	11.9
4	16	3.82 (95.6%)	1.75×10^{-1} (4.4%)	1.01×10^{-3} (0.0%)	1.76×10^{-1} (4.4%)	15.8
4	20	3.78 (94.5%)	2.17×10^{-1} (5.4%)	1.56×10^{-3} (0.0%)	2.18×10^{-1} (5.5%)	19.8
4	40	3.58 (89.6%)	4.11×10^{-1} (10.3%)	5.92×10^{-3} (0.1%)	4.17×10^{-1} (10.4%)	39.6

[a] $[Mn(II)\text{-}PICA]_T = [Mn(PICA)^+] + [Mn(PICA)_2] + [Mn(PICA)_3^-]$

Table S4. Speciation of Mn(II) (μM and the percentage) in Figure 2B and 2C.

Total [Mn]	Total [PICA]	$[Mn^{2+}]$	[MnL]	$[MnL_2]$	$[MnL_3]$	$[Mn(II)\text{-}PICA]_T$ [a]	Uncomplexed PICA
10	50	8.74 (87.4%)	1.23 (12.3%)	2.19×10^{-2} (0.2%)	1.55×10^{-5} (0.0%)	1.26 (12.6%)	48.7
100	500	40.8 (40.8%)	51.1 (51.1%)	8.06 (8.1%)	5.06×10^{-2} (0.1%)	59.2 (59.2%)	433

[a] $[Mn(II)\text{-}PICA]_T = [Mn(PICA)^+] + [Mn(PICA)_2] + [Mn(PICA)_3^-]$

Table S5. Initial first-order rate constants ($k_{initial}$) of degradation of MB by PAA-Mn(II)-PICA at initial pH 3.1–9.0 and 22°C ($R^2 > 0.94$). Reaction conditions: $[MP]_0$ = 15 μM, $[PAA]_0$ = 200 μM, $[Mn(II)]_0$ = 10 μM, $[PICA]_0$ = 50 μM.

$k_{initial}$ (min^{-1})			
**pH 3.1**	**pH 5.0**	**pH 7.0**	**pH 9.0**
$(1.65 \pm 0.20)\times10^{-3}$	$(2.30 \pm 0.15)\times10^{-1}$	$(4.10 \pm 0.32)\times10^{-1}$	$(2.41 \pm 0.20)\times10^{-1}$

Table S6. Initial first-order rate constants ($k_{initial}$) of degradation of MB by PAA-Mn(II)-PICA in the presence of various anions at initial pH 7.1 and 22°C ($R^2 > 0.88$). Reaction conditions: $[MP]_0$ = 15 μM, $[PAA]_0$ = 200 μM, $[Mn(II)]_0$ = 10 μM, $[PICA]_0$ = 50 μM, $[anions]_0$ = 10 mM.

$k_{initial}$ (min^{-1})		
**Chloride**	**Bicarbonate**	**Phosphate**
$(3.41 \pm 0.48)\times10^{-1}$	$(3.05 \pm 0.65)\times10^{-1}$	$(1.73 \pm 0.12)\times10^{-1}$

Table S7. Initial first-order rate constants ($k_{initial}$) of degradation of MPs (MB, BPA, NPX, CBZ, SMX and TMP) by PAA-Mn(II)-PICA and/or PAA-Mn(III)-PICA at initial pH 7.0 and 22°C ($R^2 > 0.89$). Reaction conditions: $[MP]_0$ = 15 μM, $[PAA]_0$ = 0 or 500 μM, [Mn(II) or Mn(III)]$_0$ = 20 μM, $[PICA]_0$ = 100 μM.

	$k_{initial}$ (min^{-1})					
**Condition**	**MB**	**BPA**	**NPX**	**CBZ**	**SMX**	**TMP**
Mn(III)-PICA	(1.03 ± 0.17)×10^{-2}	(8.10 ± 0.55)×10^{-2}	(1.65 ± 0.29)×10^{-3}	NA[a]	(2.74 ± 0.42)×10^{-3}	ND[b]
PAA-Mn(II)-PICA	(2.36 ± 0.11)×10^{0}	(6.72 ± 0.24)×10^{-1}	(1.86 ± 0.09)×10^{-1}	(1.19 ± 0.09)×10^{-1}	(1.39 ± 0.10)×10^{-1}	(1.31 ± 0.04)×10^{-1}
PAA-Mn(III)-PICA	(3.42 ± 0.14)×10^{0}	(1.07 ± 0.09)×10^{0}	(1.09 ± 0.04)×10^{0}	(2.73 ± 0.22)×10^{-1}	(1.83 ± 0.13)×10^{-1}	ND[b]

[a] Not available.

[b] Not determined.

Table S8. Initial first-order rate constants ($k_{initial}$) of degradation of MB by PAA-Mn(II)-PICA in the presence of scavengers at initial pH 5.0 and 22°C ($R^2 > 0.85$). Reaction conditions: $[MP]_0$ = 15 μM, $[PAA]_0$ = 200 μM, [Mn(II)]$_0$ = 20 μM, $[PICA]_0$ = 100 μM.

$k_{initial}$ (min^{-1})				
**No scavenger**	**50 mM TBA**	**50 mM MeOH**	**5 mM PMSO**	**N_2-purging**
(2.82 ± 0.19)×10^{-1}	(2.61 ± 0.19)×10^{-1}	(2.13 ± 0.20)×10^{0}	(2.12 ± 0.50)×10^{-3}	(2.51 ± 0.18)×10^{-1}

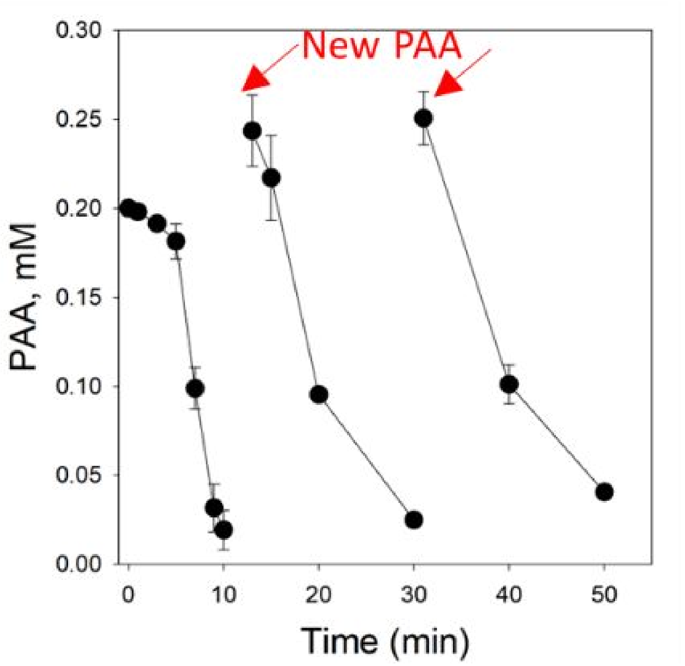

Figure S1. Continuous PAA decrease by Mn(II)-PICA. Additional PAA was added at 11 min and 31 min. (Conditions: initial pH = 5.0, $[PAA]_0$ = 200 μM, $[Mn(II)]_0$ = 20 μM, $[PICA]_0$ = 100 μM, [Mn(II)]:[PICA] ratio = 1:5).

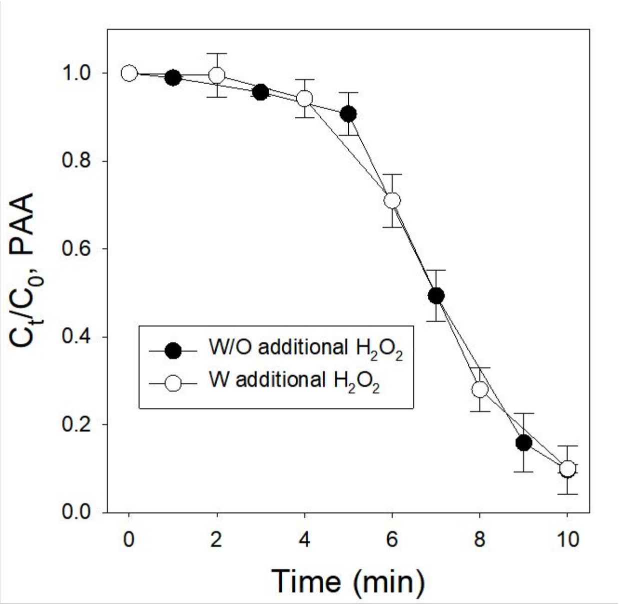

Figure S2. PAA decrease by Mn(II)-PICA with and without additional H_2O_2 input. (Conditions: initial pH = 5.0, $[PAA]_0$ = 200 μM, $[H_2O_2]_0$=84 or 184 μM (84 μM in PAA solution and additional input of 100 μM), $[Mn(II)]_0$ = 20 μM, $[PICA]_0$ = 100 μM, [Mn(II)]:[PICA] ratio = 1:5).

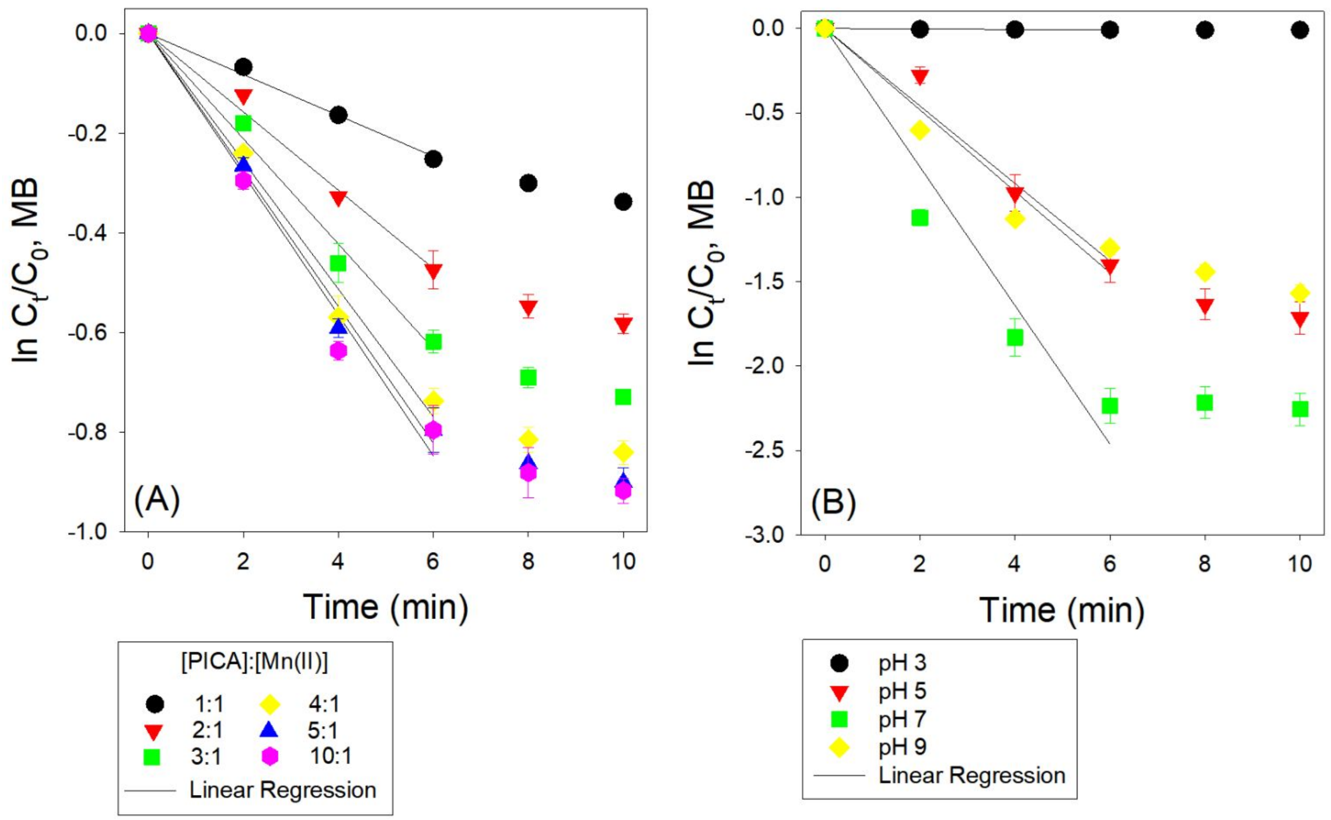

Figure S3. Initial reaction kinetics of MB by PAA-Mn(II)-PICA with (A) different molar ratio of Mn(II) to PICA (data is from Figure 2A) and (B) different pHs (data is from Figure 3A): ln(C_t/C_0) versus time relationships. Error bars of each data point indicate the standard deviation.

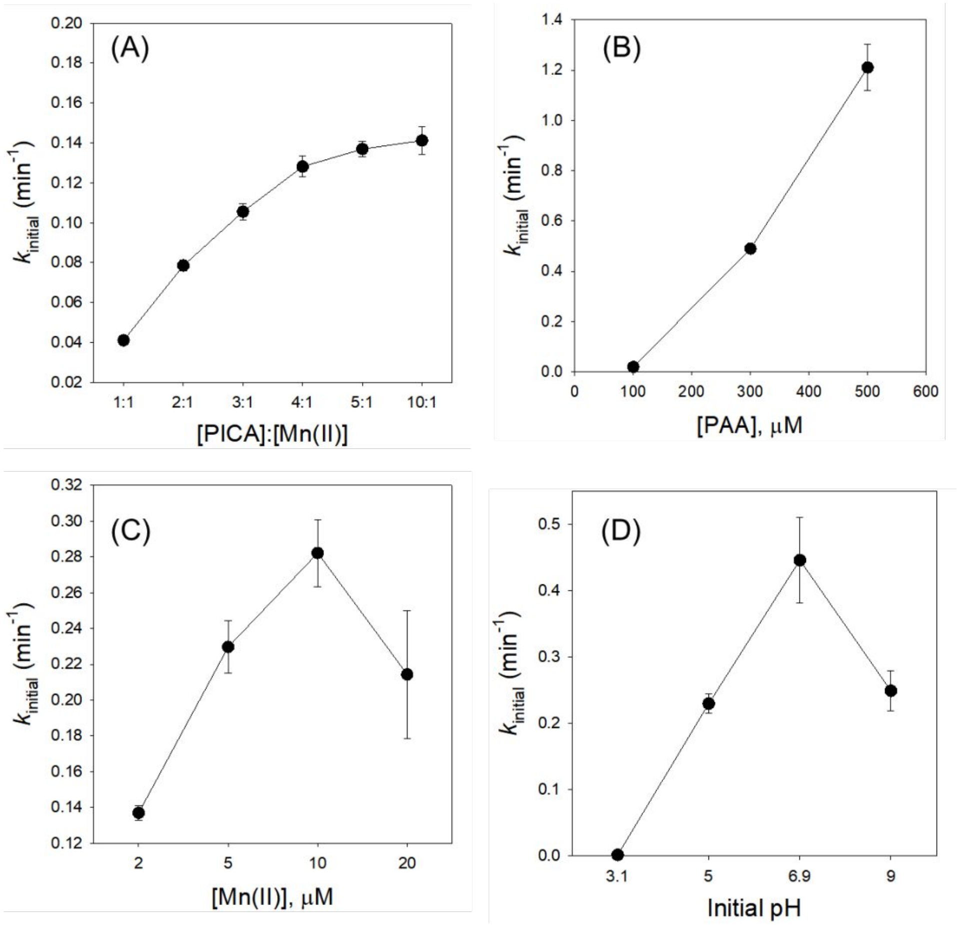

Figure S4. Initial first-order rate constants ($k_{initial}$) of degradation of MB by PAA-Mn(II)-PICA with (A) different ratio of [PICA]:[Mn(II)] (data is from Figure 2A and SI Table S2), (B) initial concentration of PAA (data is from Figure 2B and SI Table S2), (C) initial concentration of Mn(II) (data is from Figure 2C and SI Table S2) and (D) initial pH (data is from Figure 3A and SI Table S5).

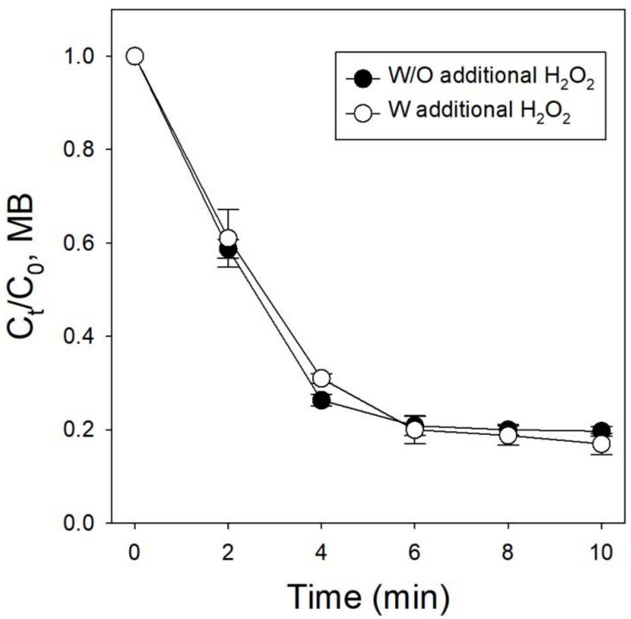

Figure S5. MB degradation by Mn(II)-PICA with and without additional H_2O_2 input. (Conditions: initial pH = 5.0, $[MB]_0$ = 15 µM, $[PAA]_0$ = 200 µM, $[H_2O_2]_0$=84 or 184 µM (84 µM in PAA solution and additional input of 100 µM), $[Mn(II)]_0$ = 20 µM, $[PICA]_0$ = 100 µM, [Mn(II)]:[PICA] ratio = 1:5).

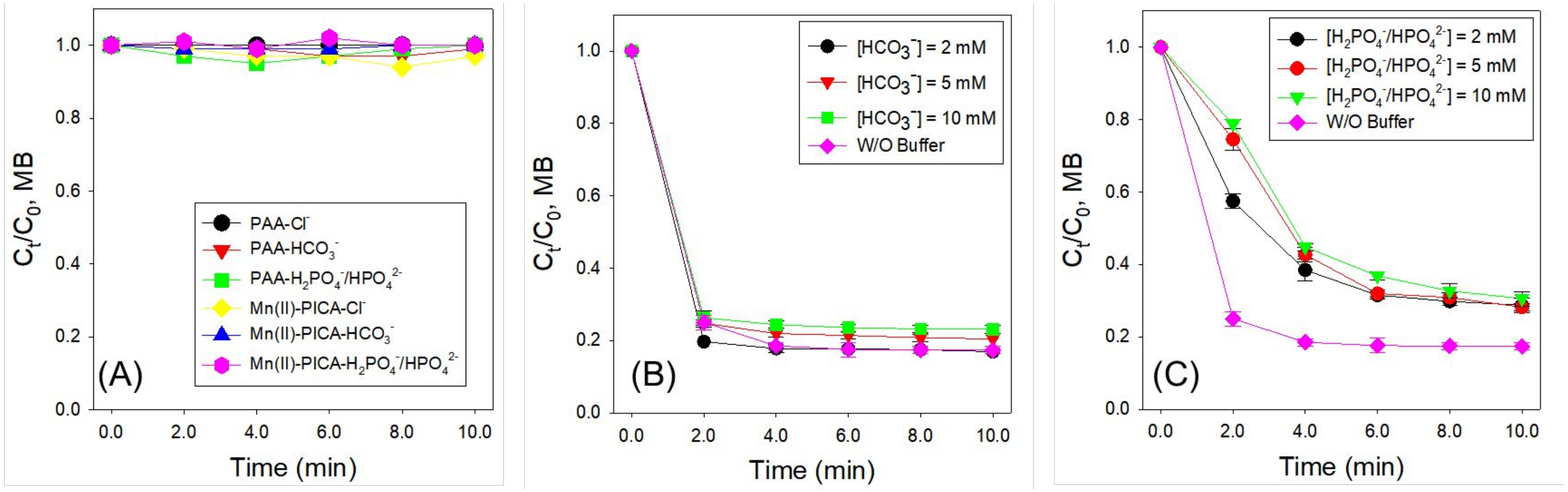

Figure S6. (A) Effects of anions on MB degradation. (B and C) Effects of different concentrations of anions on MB degradation in PAA-Mn(II)-PICA system. Conditions: (A) $[PAA]_0$ = 0 or 200 μM, $[Mn(II)]_0$ = 0 or 20 μM , $[PICA]_0$ = 0 or 100 μM, $[Cl^-$ or HCO_3^- or $H_2PO_4^-/HPO_4^{2-}]_0$ = 10 mM, initial pH = 7.1; (B and C) $[PAA]_0$ = 200 μM, $[Mn(II)]_0$ = 20 μM , $[PICA]_0$ = 100 μM, $[HCO_3^-$ or $H_2PO_4^-/HPO_4^{2-}]_0$ = 2, 5 and 10 mM, initial pH = 7.1).

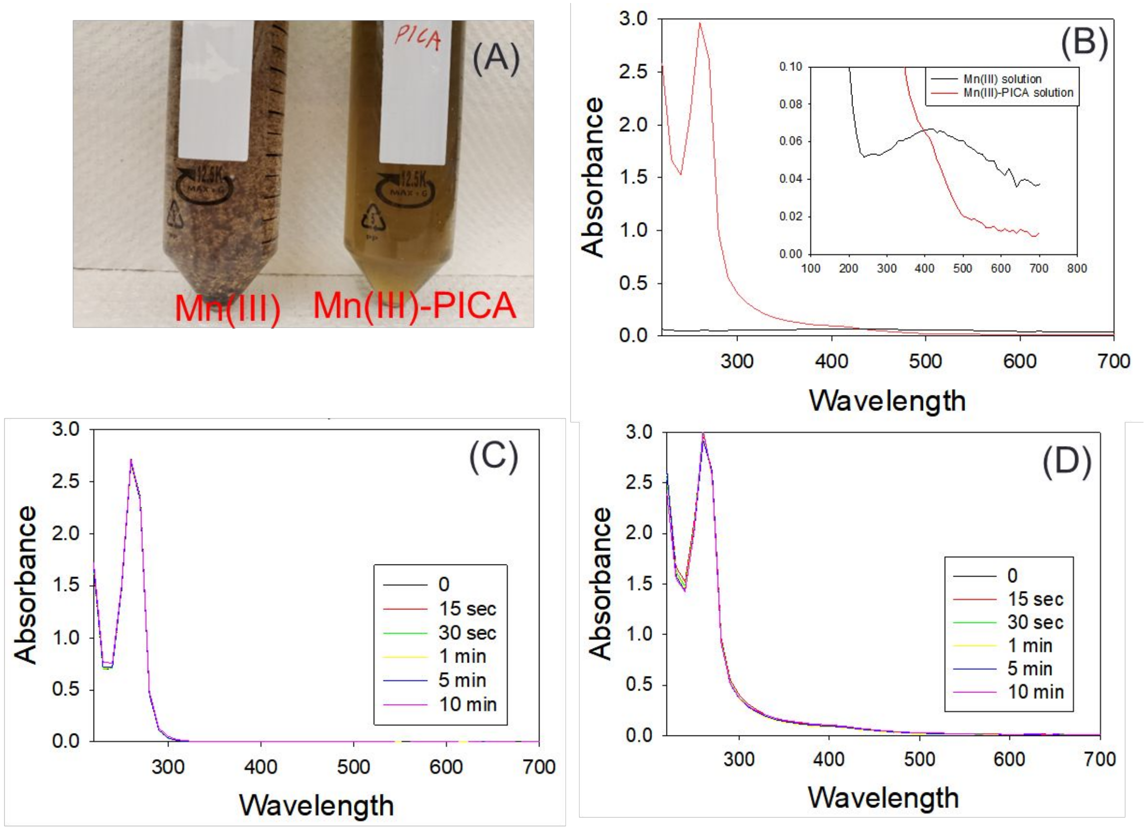

Figure S7. (A) Photograph of Mn(III) and Mn(III)-PICA solutions (pH 5, Mn(III):[PICA]=1:0 or 1:5). UV-vis spectra of (B) Mn(III) and Mn(III)-PICA, (C) PAA-Mn(II)-PICA, and (D) PAA-Mn(III)-PICA. (Conditions: [Mn(II)] or $[Mn(III)]_0$ = 100 µM, $[PICA]_0$ = 0 or 500 µM, $[PAA]_0$ = 500 M, initial pH 7.0).
